# Fuzzy Logic Tools Application to the Characterization of Stress–Strain Processes in Waste Construction Dam Geopolymers: A New Circular Mining

**DOI:** 10.3390/ma15248793

**Published:** 2022-12-09

**Authors:** Juan María Terrones-Saeta, Juan Carlos Fortes, Ana Teresa Luís, Javier Aroba, Jesús Díaz-Curiel, Emilio Romero, Jose Antonio Grande

**Affiliations:** 1 Department of Mining, Mechanical, Energetic and Civil Engineering, University of Huelva, 21819 Huelva, Spain; 2 Department of Information Technologies, Higher Technical School of Engineering, University of Huelva, 21007 Huelva, Spain; 3 Department of Energy and Fuels, School of Mines and Energy, Universidad Politécnica de Madrid, 28003 Madrid, Spain

**Keywords:** fuzzy logic, geopolymers, mining waste, circular mining, sustainability, construction materials, compressive strength

## Abstract

The ceramics industry dedicated to the manufacture of building materials is a very significant cause of environmental pollution, and various research projects are being carried out to reduce the associated environmental impact. One of the most important research lines is the generation and development of new materials, from waste, through more sustainable production processes. All of this is framed in circular mining. In this research study, geopolymers were developed with biomass bottom ashes and brick dust in order to replace the traditional ceramics used to construct bricks. For this, different families of test tubes were formed with different percentages of both residues, and their physical and mechanical properties were studied. In this way, the properties of geopolymers could be compared with traditional ceramics. In addition, in order to determine the cause–effect relationships between physical properties and compressive strength, data were processed using fuzzy logic and data mining techniques. The results showed the feasibility of geopolymers generation with biomass bottom ashes and brick dust with acceptable properties to replace conventional ceramics. In addition, the fuzzy logic analysis allowed for establishing clear and objective relationships between the physical properties and the compressive strength of the geopolymers, with the aim of developing the highest quality geopolymer.

## 1. Introduction

The material named geopolymer was developed by Joseph Davidotis in the 1970s [1]. This material is an inorganic polymer that comes from the alkaline activation of an aluminosilicate source [2]. The mixture of both components, in the presence of an aqueous medium, develops the so-called geopolymerization reaction [3].

Therefore, the shaped material has adequate strength and properties [4] for use in different fields of engineering [5], mainly in the construction [6] and mining [7] sectors. In these sectors, it has been used in various studies as a substitute for cement [8], concrete [9] and even mortar [10], thanks to its properties of fire resistance [11], stability at high temperatures [12], compatibility with structural steel [13], etc.

In addition, most of the developed studies use residues from other activities as raw materials [14,15] for the conformation of the geopolymer. Therefore, the environmental impact associated with its manufacture, as well as the carbon footprint produced, is very reduced [16,17]. There are several materials that have been used as a source of aluminosilicates for geopolymers, these being mainly carbon float [18], metakaolin [19], metallurgical slag [20] and glass [21]. However, as an alkaline activator, sodium hydroxide [22] or potassium hydroxide [23] are usually used, both commercial products.

It is because of this that the geopolymer is currently one of the most sustainable materials existent, with a greater impact on the future [24]; once it uses wastes as raw materials [P], its conformation process produces low CO_2_ emissions [25] and, in addition, it has similar characteristics to the conventional construction materials used [26].

However, its use, as commented above, is mainly based on substitutes of cement or concrete, unlike this research. In this work, we proceed to the generation and testing of the geopolymer as a substitute element for ceramic materials commonly used for bricks and tiles since these products are produced in large quantities and have a significant environmental impact [27]. In this way, we generated an economic asset (bricks) from an environmental liability (waste from the quarry-ceramic industry).

The manufacture of bricks from the traditional ceramic industry extracts huge quantities of clay, with the consequent economical cost and environmental impacts producing, in turn, greenhouse gas emissions in the sintering process that is carried out at high temperatures, usually around 950 °C [28].

Therefore, the use of geopolymers with the use of waste, as presented in this research study, would largely avoid the environmental effects caused by traditional ceramics since the mining tasks of clay extraction would be eliminated, and more energetically optimized manufacturing processes would be developed [29].

In addition, the use of wastes for geopolymers manufacture as substitutes for traditional ceramics prevents the deposition of these by-products in large landfills, giving them a new useful life and, in turn, eliminating the extracting works of new raw materials [30]. It is, therefore, a clear example of the new circular mining [31], in which the wastes from an industrial/mining activity are the raw materials for other activities, thus closing the materials flow and avoiding unwanted landfills by transforming environmental liabilities into new economic assets. Therefore, the use of geopolymers made from wastes as raw materials would significantly reduce the environmental impact caused by this sector.

For the development of these substitute geopolymers of ceramic materials for construction, mainly bricks, two types of wastes were used. On the one hand, and as a source of aluminosilicates, brick dust produced in the ceramic industry was used [32]. On the other hand, and as an alkaline activator, opposite to other researchers who activate with potassium or calcium hydroxides, biomass bottom ashes from the almond shells and alperujo (residue from the olive oil agri-food industry) combustion was used. These ashes, produced in electricity production plants, do not currently have use and, in most cases, are deposited in landfills. However, their high potassium oxides content, due to their formation process, make them an ideal element for use in geopolymers formation as an alkaline activator [33].

Therefore, in this research study, geopolymers were developed with brick dust and biomass bottom ashes to evaluate their physical and mechanical properties, with the sole purpose of determining their possible use as substitutes for ceramic materials of bricks in the construction sector. For this, different families of test tubes were formed with different percentages of both residues, and subsequently, their physical and mechanical properties were evaluated.

The test results were initially analyzed with descriptive statistics to assess the results’ quality and, subsequently, with fuzzy logic tools.

The analysis of the data by applying a fuzzy logic and data mining tools methodology (PreFuRGe) [34] reflected the existence cause–effect relationships [35] essential to, on the one hand, understanding the geopolymerization process and, on the other hand, determine which physical properties influence the geopolymer formation with the highest possible compressive strength, which is desirable.

The results of this research study showed that it was feasible to make geopolymers with brick dust and biomass bottom ashes, reflecting the usefulness of fuzzy logic in establishing the relationships between the different physical and mechanical properties of the formed geopolymers.

Therefore, the novelty of this study derives from the fact that not only a sustainable material substitute for traditional ceramics was developed for the construction sector, but also the data were analyzed in depth to determine the relationships between the different physical properties, being able to predict the structural behavior of the geopolymer based on its basic measured properties. This fact is unusual and impossible to relate to traditional classical statistics.

Consequently, the central objective of this work is specified in an operation model proposal of stress–strain processes for geopolymers by applying a fuzzy logic and data mining-based methodology, using datasets related to stresses, rupture limit and physical properties of the geopolymer.

## 2. Materials and Methods

In this section, firstly, the properties and nature of the wastes used for the formation of geopolymers were detailed, with brick dust and biomass bottom ash being the wastes of choice. Then, the methodology for shaping the geopolymers is described, as well as the procedure followed for the different tests to determine the physical and mechanical properties. Finally, the statistical methods were described, on the one hand, to determine the quality of the data obtained in the tests and, on the other hand, to evaluate, through fuzzy logic, the different relationships between the multiple variables characterized by the geopolymers.

### 2.1. Materials

The materials used in this study were industrial wastes. Therefore, usable material for construction was created with 100% waste, with the environmental advantages that this entails. Brick dust is the perfect source of aluminosilicates for forming geopolymers. This brick powder was alkali-activated with biomass bottom ashes through the geopolymerization process. It should be noted that biomass bottom ashes are derived from the combustion of almond shells and “alperujo” for energy production. Consequently, they have physical and chemical properties that are constant over time, which makes them useful to use in geopolymers, as is the case of brick dust.

#### 2.1.1. Brick Dust

Brick dust derives from the ceramic industry located in the south of Spain, more specifically in Linares (Andalusia). This residue is inherently obtained in the production of bricks or tiles for the construction sector; once after the sintering process, there are different pieces that do not have the appropriate dimensions or that break during the process. Therefore, this material is removed and crushed to occupy as less space as possible, used anecdotally for some low-cost functions, and its properties are not optimized for use as raw material in higher-quality materials elaboration [36].

It should be noted that, as this brick powder is produced from already sintered ceramic pieces, its physical and chemical properties are not at all similar to those of conventional clay.

The most notable characteristics of this waste are its density, around 2.54 t/m^3^, and its particle size, which is less than 200 µm and greater than 40 µm. On the other hand, the chemical composition of brick dust is mainly silicon, aluminum, calcium, iron, potassium and magnesium. At the same time, it should be noted that the percentage of carbon, nitrogen, hydrogen and sulfur is very low, something to be expected taking into account the sintering process from which it derives and, consequently, the ignition loss of the sample is 1.74%, which is, very low. Therefore, and due to the silicon percentage of 27.32% and aluminum of 8.16%, it can be said that this residue has an ideal chemical composition to form geopolymers, as well as a very suitable particle size.

#### 2.1.2. Biomass Bottom Ashes

The biomass bottom ashes, hereinafter BBA, used for the development of geopolymers as an alkaline activator, are also derived from the industries located in the south of Spain (Linares, Andalusia) for electricity generation. More specifically, these ashes come from the combustion of almond shells and “alperujo” (a by-product obtained in the oil agri-food industry), so their chemical composition and properties are unalterable over time [37].

These biomass bottom ashes have a very similar density to brick dust, 2.65 t/m^3^, so their compatibility is ideal for the homogenization and mixing process. In addition, the particle size is very similar, between 10 and 200 µm. The ashes’ chemical composition is suitable for use as an alkaline activator since it has a large percentage of potassium, silicon, calcium, phosphorus, magnesium, aluminum and iron oxides. In addition, the ignition loss is 8.16%, showing a very low percentage of carbon and hydrogen that demonstrates the almost inexistence of unburned organic matter [38].

### 2.2. Methodology

This section details the methodology followed for shaping the geopolymers and the execution of the different tests that determine their physical and mechanical properties. At the same time, the principles and tools used for the analysis of all the obtained data from the characterization of geopolymers are shown.

#### 2.2.1. Shaping and Testing of the Different Families of Geopolymer’s Test Tubes

For the geopolymer shaping process, all raw materials, brick dust and biomass bottom ashes were dried for 24 h at 105 ± 5 °C. With the aforementioned residues, the manufacture of the different test tube families with different percentages of them was performed. First, a 100% brick dust family and, subsequently, other families with increasing percentages of biomass bottom ashes of 10% in decreasing percentage of brick dust. The second family was made up of 10% biomass bottom ashes and 90% brick dust, the third with 20% biomass bottom ashes and 80% brick dust, etc., until reaching the 100% biomass bottom ashes and 0% brick dust. It should be noted that both made up of 100% biomass bottom ashes and the one with 100% brick dust were discarded from the statistical analysis since the geopolymerization process did not occur because a source of aluminosilicates with an alkaline activator did not combine. Therefore, these families were only made in order to confirm that the geopolymerization process actually took place once the detailed families did not have adequate physical properties or mechanical resistance. The different families of shaped test tubes are shown in Table 1.

The shaping process of the 6 test tubes for each family was always the same to avoid errors. First, both residues were weighed in the appropriate proportions and mixed. Then, 20% of water was added to activate the geopolymerization process, and the mixture was homogenized again. The mixture obtained from the different families was transferred to a metallic matrix of 60 mm × 30 mm to exert a pressure of 30 MPa. Once the test tubes were formed, they were removed from the matrix and deposited for 24 h at room temperature (20 ± 2 °C) and then at 90 ± 2 °C for 24 h. After this drying period, to facilitate geopolymerization, the test tubes of all families were measured, weighed and immersed in a circulating water bath at a temperature of 20 ± 2 °C. The purposes of this process were, on the one hand, to eliminate all those chemical compounds that have not reacted in the primary geopolymerization process and, on the other hand, to try to react to those compounds that demanded more water in the initial geopolymerization process. Finally, all the specimens were dried again for 24 h at a temperature of 90 ± 2 °C and were subsequently measured and weighed again to determine the mass loss and the linear contraction that they suffered, according to the UNE-EN 772-16 standard.

Next, and once the geopolymers were dry, the water absorption by capillarity was determined according to the UNE-EN 772-11 standard. This test is based on the determination of the mass variation experienced by the geopolymer before and after its submersion for 60 ± 1 s in 5 mm of water. In this way, the capillarity of the structure could be determined.

Subsequently, the different samples of geopolymers from each family were immersed in water at room temperature (20 ± 2 °C) for 24 h to evaluate the cold water absorption according to the UNE-EN 772-21 standard. In this way, the quality of geopolymers could be evaluated for their use in elements found outdoors once those elements are more affected by climatic conditions.

In turn, the boiling water absorption test was carried out after the previous test, according to the UNE-EN 772-7 standard, which allowed the structure of the geopolymer to be appreciated. Therefore, it had a high porosity or, on the contrary, it was very compact. This test, together with the open porosity and bulk density test (UNE-EN 772-4), was carried out in the same way, immersing the test tubes in boiling water to later measure their submerged mass in water and their mass with dry saturated surface.

Finally, the mechanical properties, which obviously must have a construction material and the geopolymer under study, were evaluated through the compression test, carried out according to the UNE-EN 772-1 standard. This test is essential, not only because the geopolymer as a construction material must have an intrinsic resistance equal to that of a conventional ceramic material but also because it reflects the quality of the formed geopolymer and the feasibility of using both residues to produce geopolymers. Therefore, this mechanical property, as it is detailed later, is the one that was taken as a consequence of all the statistical analysis through fuzzy logic.

It should be noted that once the geopolymer formed with almond shell and biomass bottom ashes wished to replace conventional traditional ceramic materials, different test tubes were manufactured with red clay with the same procedure that was described for geopolymers. This process only differed in sintering, which was carried out at 950 ± 5 °C for 1 h. Therefore, in this study, it was possible to evaluate the existing differences between the physical and mechanical properties detailed in the geopolymers with the traditional ceramic material.

#### 2.2.2. Statistical Summary

Once the different families of test tubes were formed, according to the procedure detailed above, and the necessary physical and mechanical tests were carried out, the data were analyzed using the Statgraphics Centurion XVI software version 16.2.04 from Statgraphics Technologies, Inc. (The Plains, VA, USA).

This data analysis, based on descriptive statistics, made it possible to process and analyze the data for subsequent treatment with fuzzy logic, determining their quality, as well as possible errors that could have been made during the methodology following [39]. Thus, with this tool, it was possible to analyze the quality of the results through descriptive techniques for further analysis by using fuzzy logic techniques.

Therefore, with the Statgraphics Centurion XVI software version 16.2.04 from Statgraphics Technologies, Inc. (The Plains, VA, USA) software, parameters of the obtained data in the geopolymer tests were analyzed, such as the average, the standard deviation, the minimum and the maximum, summarizing all these values in the statistical summary [40].

In this way, it was possible to make a first approximation to the knowledge of the physical and mechanical properties of geopolymers in comparison with traditional ceramics once average values of these characteristics were obtained for their possible evaluation. In addition, parameters such as standard deviation allowed knowing the suitability of the data in order to obtain an interpretation that is as objective as possible to determine the feasibility of manufacturing geopolymers made from brick dust and biomass bottom ashes.

#### 2.2.3. Data Mining and Fuzzy Logic (PreFuRGe)

Fuzzy logic, unlike classical statistics, allows data to be interpreted and related to each other through different cause-and-effect links in a much more real way. This is due to the fact that classical statistics establish relationships that can be denominated as yes or no, 0 or 1, while fuzzy logic establishes relationships that are evaluated numerically between 0 and 1 [41].

In this way, a large data mass can be analyzed objectively and determine if the relationship between the different physical and mechanical properties, as in this case, is high, low, total or non-existent, showing a wide dependence field between variables that was totally ignored by classical statistics. It is, therefore, a way of analyzing data developed by many researchers that have been successfully applied in various fields [42,43] since it allows analyzing a large data mass objectively, quickly, intuitively and obtaining relationships between numerically detailed variables similar to the appreciation that a human expert would do. Moreover, in [44,45], it was observed that other fuzzy logic methodologies had been used successfully in similar problems, with satisfactory results.

In addition, this type of statistics, based on heuristic rules, allows defining an antecedent to further determine a consequent. In this way, both are related since if the antecedent increases, the consequent will act according to the established relationship and vice versa.

**Figure 1:**
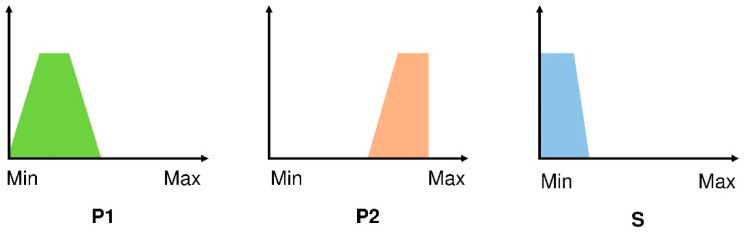
Example of fuzzy rule (antecedents: P1, P2 and consequent: S).

All this can be easily explained to the reader in the following example of a fuzzy rule shown in Figure 1.

The interpretation in the natural language of the fuzzy rule represented in Figure 1 is: IF P1 is bigger than small and lower than average, and if P2 is big, then S is small. These assessments could not be made with classical statistics since here the field that can be covered by the antecedents P1, P2 and the consequent S is detailed; that is, the answer is not unique as in classical statistics Yes or No, 0 or 1.

##### PreFuRGe Methodology (Predictive Fuzzy Rules Generator)

PreFuRGe [34] is a soft computing tool based on fuzzy logic addressed with the methodology proposed by Sugeno in [46] and based on the FCM (fuzzy C-means) algorithm [47], designed to work with quantitative datasets with the objective of generating fuzzy systems made up of a set of IF-THEN fuzzy rules as follows: IF v1 ∈ C1 AND v2 ∈ C2… THEN s ∈ CS2, where x = (v1, v2,…, vn) ∈ Rn are input variables (antecedents), C1, C2, … Cn are n fuzzy sets, s ∈ ℜ is the output variable (consequent) and CS2 is a fuzzy set for this variable.

The main steps of the PreFurGe methodology, once the datasets to analyses have been pre-processed to filter outliers, noise, etc., are:

(1) The expert has to decide the goal parameters to study (consequents) to discover their relationship with the rest of the variables (antecedents).
(2) The datasets are processed, starting with the classification of the goal variables (consequents) in an appropriate set of fuzzy clusters [37,46,48]. The optimum number of fuzzy clusters obtained will determine the number of fuzzy rules that conform to the final fuzzy system.
(3) Each fuzzy cluster of the previous step is projected onto the space of the antecedents [46] so that a fuzzy set is calculated for each antecedent variable in each fuzzy cluster.
(4) The obtained fuzzy rules are determined with numerical precision and also provide a graphical way (Figure 1) that it is possible to interpret in natural language, where:
  - The fuzzy set calculated for each variable (antecedent or consequent) is approximated by a trapezium (to facilitate the interpretation);
  - The values of each variable are represented in the x-axis;
  - The membership grade to each cluster is represented in the y-axis.

The obtained fuzzy rules in the generated fuzzy system perfectly could be used as a fuzzy inference system to predict the expected value of the consequent from a set of antecedent values. However, the main goal of this study is to obtain a qualitative model that describes the relation of the variable “compressive strength” with the rest of the parameters (Section 3.2) and not to predict values. Therefore, in this work, it was not necessary to define a defuzzification method or the type of aggregation of rules.

It should be noted that, as can be seen in the previous example, the results of the statistical analysis through fuzzy logic are represented in graphs (fuzzy rules). These fuzzy rules reflect the antecedents and consequents in a clear and simple way, as well as numerically. In this way, after analyzing all the data, it is possible to obtain this type of graph that greatly simplifies the work of the reader; with a simple glance, it is possible to know the cause–effect relationships that take place between the different variables.

In this study, the different variables related to the physical properties defined in the methodology were those that were taken as antecedents, just being the compressive strength the consequent. This analysis hypothesis was based on a fundamental principle, the resistance of the formed geopolymer. For this reason, resistance was taken as a consequence since it was desired to obtain a resistant material to be a substitute for ceramic materials for construction. In this way, it was possible to evaluate what variation in the physical properties occurred with the increase or decrease in the resistance, as well as how these physical properties should be to obtain the greatest geopolymer resistance.

This study was carried out by applying fuzzy logic and data mining techniques (PreFuRGe) [34]. This methodology was already used for data analysis in different fields and with a multitude of consequent and antecedent variables, thus allowing for determining the cause–effect relationships occurring between various variables in a simple, graphic and fast way. Obviously, all these qualitative and quantitative relationships derived from the analysis through fuzzy logic and data mining (PreFuRGe) are graphically represented as a set of fuzzy rules and are studied in this work. Thus, it was possible to define which physical properties should increase or decrease to achieve a greater resistance in the formed brick dust and biomass bottom ashes geopolymers and vice versa.

## 3. Results and Discussion

**Figure 2:**
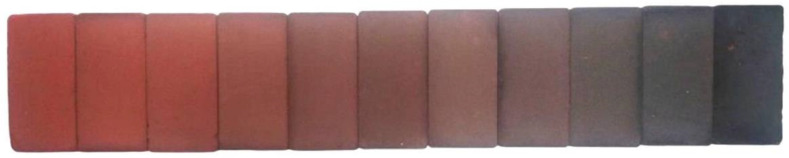
Image of the different families of samples formed with different percentages of brick dust and biomass bottom ash.

The results of the tests, as well as their discussion, appear in this section in the same order in which they have been presented in the methodology. These results from the different conformed geopolymer samples (Figure 2) allow us to obtain a series of partial conclusions that lead to the final conclusion, which is the evaluation of the viability of the manufacture of geopolymers with brick dust and biomass bottom ash as substitutes for traditional ceramic materials. The image of the different families of specimens is shown in Figure 2.

### 3.1. Statistical Summary

The statistical summary of the different data analyzed through descriptive statistics with the Statgraphics Centurion XVI software version 16.2.04 from Statgraphics Technologies, Inc. (Virginia, USA) allows a first approximation of the information carried by the variables, as well as knowing the quality of the results and the average values of the different physical and mechanical properties. Therefore, these results are essential for a better further interpretation of the fuzzy logic analysis and, in turn, essential to determine the similarity or difference of the geopolymers formed with biomass bottom ashes and brick dust with traditional ceramics. The statistical summary is shown in Table 2.

As detailed in Table 2, the first of the physical properties analyzed was weight loss. The statistical summary shows that the data show discrete values with the exception of the variance % for the linear shrinkage and the compressive strength, phenomena that are explained by the fuzzy rules of this work and are the response of the geopolymer. The average value of the weight loss in the formed geopolymers was 6.415%. Due to the fact that the minimum and maximum weight loss of the geopolymers after the detailed geopolymerization process is 2.696% and 12.313%, it can be stated that this physical property is very similar in traditional ceramics made with clay since these obtain a 9.536% weight loss after the sintering process.

On the other hand, the linear shrinkage of the geopolymer families developed under the described conditions shows a 0.157% average linear shrinkage. This value is much lower than that obtained in traditional ceramics with clay (2.714%). However, it clearly represents that the sintering process of ceramics creates a much more closed structure since it is carried out at high temperatures. This fact obviously determines the subsequent properties of the geopolymer.

In turn, the water absorption by capillarity had an average value of 2967 g/m^2^min in the families of geopolymers formed with brick dust and biomass bottom ashes. On the other hand, the water absorption by capillarity of the ceramics formed with the same methodology described above has a water absorption by capillarity of 1700 g/m^2^min, which shows that the geopolymer has a more open and interconnected pore structure that is capable of absorbing a higher percentage of water. However, the average values of water absorption by capillarity are acceptable according to European regulations for ceramic materials used in construction.

The cold water absorption tests, directly related to the boiling water absorption, reflected average values of both properties of 18.336% and 19.152%, respectively. For once, a cold water absorption of 12.348% and a boiling water absorption of 13.574% existed for traditional ceramics made with clay. Therefore, these results, together with the previous property of water absorption by capillarity, demonstrate that the formed geopolymers have a more open structure and a greater number of interconnected pores. This property of geopolymers, even though it negatively affects the mechanical resistance of the material, provides very interesting properties for the material, such as thermal and acoustic isolation [49].

In turn, the average values of open porosity and bulk density for the formed geopolymers were 29.828% and 1.648 t/m^3^, respectively. Values, as expected from what was detailed above, are lower than those obtained in traditional ceramics. The ceramics made with red clay obtained an average open porosity of 24.893% and an apparent density of 1.987 t/m^3^. Therefore, it can be confirmed that traditional clay-formed ceramics have a much more compact structure, with less porosity, higher density and less capacity for water absorption than geopolymers formed with brick dust and biomass bottom ashes. However, these characteristics of the formed geopolymers can become interesting as long as their mechanical resistance requirements are accomplished; therefore, the compression test was carried out.

Finally, the geopolymer compression test reflected an average value of 33.501 MPa, lower than that obtained for traditional ceramics made with the same process of 42.361 MPa, but higher than the minimum limit set (10 MPa) by the regulations for this type of material. Therefore, it can be concluded that the results obtained from the physical and mechanical properties test of geopolymers are acceptable according to the regulations of ceramics for construction.

It is worth noting that the maximum and minimum values of the different properties, as well as the averages and the standard deviation, are acceptable according to the methodology followed to obtain the different families of geopolymers and for their further analysis with fuzzy logic.

### 3.2. Qualitative Results: Fuzzy Rule-Based Systems

**Figure 3:**
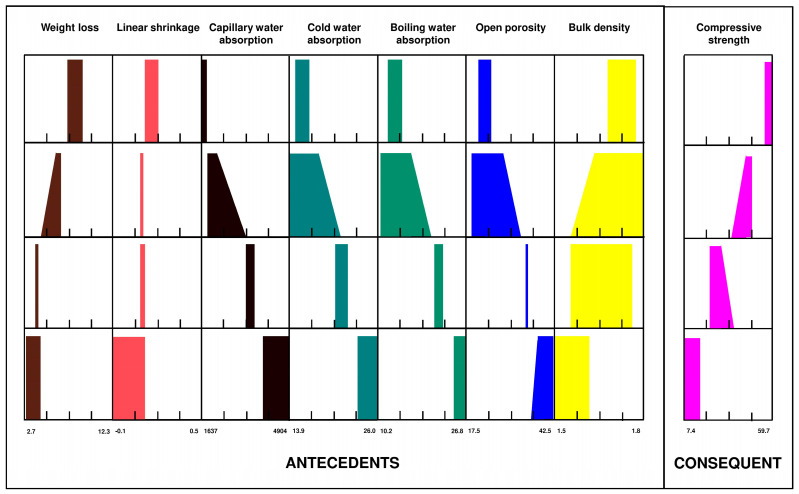
Fuzzy rules (compression strength as consequent).

The biomass bottom ashes and brick dust formed geopolymer will serve as ceramic material; therefore, it is necessary that this mechanical property be evaluated, as well as the interdependence of other physical properties with the resistance. The results for the different families of geopolymers formed with brick dust and biomass bottom ash are represented in Figure 3.

According to the results obtained from the statistical summary of variables and for a better understanding of the processes and results, the same datasets have been processed by using the proposed fuzzy logic methodology [34], which allowed for establishing the potential cause–effect relationships between the different variables under study, taking compressive strength as a consequent and the rest of the variables as antecedents in the obtained fuzzy rules (Figure 3).

In the first rule (upper row) of the obtained fuzzy rules (Figure 2), when compressive strength takes extremely high values, it can be seen how the bulk density shows high to extremely high values. At the same time, for the same compression value, the open porosity, cold water absorption and boiling water absorption should present low to extremely low values, identical behavior for these three variables. In turn, the absorption of water by capillarity takes very concentrated values in the extremely low range, at the same time that the mass loss and the linear contraction take concentrated values in medium ranges.

The fact stated in the previous paragraph can be validated if the mechanical behavior of the geopolymer is known, which means a higher apparent density will condition, under equal conditions, a geopolymer that is more resistant to compression, which, in turn, will have a lower porosity. Therefore, if the geopolymer has a lower open porosity, the absorption of water by capillarity, cold water absorption and boiling water absorption must also be lower due to the concept of porosity relative to the volume of holes compared to the total volume of the sample in the study. In addition, and due to the fact that the geopolymer was manufactured with waste, a greater weight loss implies the disappearance of residual substances present in the spaces of the structure, obtaining a greater resistance to compression. It should be noted that the linear contraction of the geopolymer during the geopolymerization process also implies a higher apparent density once its volume is reduced for the same mass and, consequently, a higher compressive strength.

This phenomenon, although evident, is validated by the numerical relationships collected in the data mass that shape the matrix from which the set of fuzzy rules in Figure 3 is obtained. Otherwise, it would have been difficult to establish the cause–effect relationships that occur between physical properties and compressive strength in the forming process.

Consequently, and as is evident in Figure 3, it can be stated that an increase in weight loss during the geopolymerization process develops a geopolymer with greater mechanical resistance since, as mentioned, residual compounds of the geopolymer are eliminated, which impairs the geopolymerization process.

In turn, a higher linear shrinkage directly causes a higher bulk density, so the compressive strength of the geopolymer increases. This fact is easily visible in Figure 3; note how as one variable increases from rule to rule (row to row), so does the other and vice versa.

On the other hand, it must be stated that a greater porosity conditioned by greater absorption of water by capillarity, cold water absorption and boiling water absorption determines a lower resistance to compression. This fact is obvious once a higher pore index is capable of absorbing a higher water percentage and, in turn, lowering the apparent density that determines the compressive strength.

**Figure 4:**
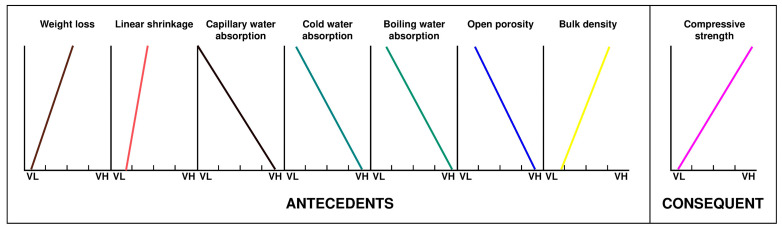
Trending lines of the different variables throughout the discourse universe for each of the 4 fuzzy rules from the “centers of gravity” of the figures corresponding to the values “extreme-high” and “extreme-low”.

If Figure 3 is simplified to observe the trends of each variable based on the section imposed by the consequent in 4 values from extremely low to extremely high, Figure 4 is obtained.

Figure 4 globally represents the global behavior of the different variables or physical properties based on the consequent compressive strength. This figure was designed taking into account the antecedent’s variations as a function of the resistance, simplifying Figure 3 and directly showing the relationships occurring between the antecedents and the consequent in a global way for all the variables as a whole, which allows us to “bird’s-eye view” to check the proportionality, direct or inverse of some variables with respect to others.

Essentially, Figure 4 allows for immediately observing how the different variables increase or decrease as the consequent does for the four sections imposed by the compressive strength itself.

Therefore, it can be seen that weight loss, linear shrinkage and apparent density are directly proportional to the increase in compressive strength, something that was expected if the geopolymerization process and the characteristics of the mining waste used are taken into account.

On the other hand, the open porosity, the absorption of water by capillarity, the cold water absorption and the boiling water absorption are inversely proportional to the compressive strength of the geopolymer; that is, if the variables mentioned decrease, a higher compressive strength geopolymer is developed. Obviously, this relationship between variables has an explanation since if there is a greater open porosity, there is also a greater water absorption, demonstrating that the geopolymer has a more open and less compact structure that makes it less resistant.

However, it should be noted that maximum resistance is not always necessary in the construction sector, but other properties, such as thermal or acoustic isolation, are also attractive, directly conditioned by a greater porosity and a lower apparent density. For this reason, complying with the resistance limit established by the regulations for ceramic construction materials of 10 MPa, various geopolymers can be obtained with specific characteristics of acoustic isolation, resistance, exposure to the elements, etc., with only the combination of various percentages of brick dust and biomass bottom ashes. In this way, it is achieved with both waste materials and various specific characteristics for different functions that are going to develop in their useful life.

Consequently, the novelty of the application of fuzzy logic techniques for the characterization tests of geopolymer material allows the percentages of waste contributions to be calibrated in order to achieve a balance between resistance, porosity, capillarity, acoustic isolation, etc., to cover the different market needs, always within limits imposed by the regulations.

## 4. Conclusions

Based on the results obtained after the tests and the data treatment of the variables that characterize the different families of geopolymers, made up of biomass bottom ashes and brick dust, the following partial conclusions could be formulated, which, in turn, leads to the definition of the final conclusion, covering the central objective of this work:

- The statistical summary of variables showed the possibility of making geopolymers with brick dust and biomass bottom ashes that have acceptable physical and mechanical properties for their use as traditional ceramics since the results of the tests carried out are similar to those of ceramics and respect the current regulations.
- Statistical analysis showed that the conformed geopolymers had a weight loss similar to traditional ceramics. However, capillary water absorption, cold water absorption, boiling water absorption and open porosity were higher in geopolymers than in traditional ceramics, obtaining, in turn, a lower linear contraction, a lower density and, consequently, a lower compressive strength of the geopolymers.
- The use of fuzzy-logic techniques and data mining was a pioneer in this work for the characterization of geopolymers, revealing that it is an effective and highly defining tool of cause–effect relationships in the search for a system functioning model subjected to the different scenarios imposed by the variables.
- The data treatment with fuzzy logic techniques allowed the observation of how a greater open porosity of the formed geopolymers implied a greater water absorption by capillarity, a greater absorption of cold water and a greater absorption of boiling water, producing, consequently, lower resistance to compression. In addition, the increase in linear shrinkage produced an increase in the apparent density, which obviously leads to a higher compressive strength.
- On the other hand, the analysis with fuzzy logic showed that the decrease in mass loss was directly related to the decrease in the compressive strength of the geopolymer once a greater mass loss is due to the elimination during the geopolymerization process of all those superfluous chemical compounds that do not help to the geopolymer formation process.

Finally, and based on the partial conclusions detailed above, it can be concluded that it was possible to form geopolymers with brick dust and biomass bottom ashes from the combustion of almond shells and “alperujo”, as substitutes for ceramics usually used as construction materials, once these geopolymers have acceptable physical and mechanical properties according to the regulations. On the other hand, it should be noted that the analysis of the results of the different geopolymer tests through data mining and fuzzy-logic techniques made it possible to clearly establish the relationships between the various physical properties and compressive strength in a clear and objective way, allowing to obtain a wide range of materials with specific properties with just a combination of different percentages of both residues.

## Figures and Tables

**Table 1:** Families made up of geopolymers with different percentages of brick dust and biomass bottom ashes.

Test Tubes Families	% Brick Dust	% BBA
1	100	0
2	90	10
3	80	20
4	70	30
5	60	40
6	50	50
7	40	60
8	30	70
9	20	80
10	10	90
11	0	100

**Table 2:** Statistical summary of the data obtained from the tests to determine the physical and mechanical properties of geopolymers.

Variables	Average	Standard Deviation	% Variance	Minimum	Maximum
Weight loss, %	6.415	3.062	47.725	2.696	12.313
Linear shrinkage, %	0.157	0.131	83.683	−0.063	0.462
Capillary water absorption, (g/m^2^min)	2966.169	1128.330	38.033	1636.612	4903.865
Cold water absorption, %	18.336	5.996	32.699	10.210	26.749
Boiling water absorption, %	19.152	4.033	21.057	13.931	25.986
Open porosity, %	29.828	8.640	28.967	17.468	42.458
Bulk density, g/cm^3^	1.648	0.072	4.374	1.524	1.776
Compressive strength, MPa	33.501	17.996	53.719	7.429	59.738

## Data Availability

Data are contained within the article.
